# In Vitro Model of Neuroinflammation: Efficacy of Cannabigerol, a Non-Psychoactive Cannabinoid

**DOI:** 10.3390/ijms19071992

**Published:** 2018-07-08

**Authors:** Agnese Gugliandolo, Federica Pollastro, Gianpaolo Grassi, Placido Bramanti, Emanuela Mazzon

**Affiliations:** 1 IRCCS Centro Neurolesi “Bonino Pulejo”, 98124 Messina, Italy; agnesegugli@hotmail.it (A.G.); placido.bramanti@irccsme.it (P.B.); 2 Department of Pharmaceutical Sciences, University of Eastern Piedmont “Amedeo Avogadro”, 28100 Novara, Italy; federica.pollastro@uniupo.it; 3 Research Centre for Industrial Crops, Council for Agricultural Research and Economics (CREA-CIN), 45100 Rovigo, Italy; giampaolo.grassi@gmail.com

**Keywords:** cannabigerol, *Cannabis sativa*, neuroinflammation, oxidative stress, phytocannabinoid

## Abstract

Inflammation and oxidative stress play main roles in neurodegeneration. Interestingly, different natural compounds may be able to exert neuroprotective actions against inflammation and oxidative stress, protecting from neuronal cell loss. Among these natural sources, *Cannabis sativa* represents a reservoir of compounds exerting beneficial properties, including cannabigerol (CBG), whose antioxidant properties have already been demonstrated in macrophages. Here, we aimed to evaluate the ability of CBG to protect NSC-34 motor neurons against the toxicity induced from the medium of LPS-stimulated RAW 264.7 macrophages. Using MTT assay, we observed that CBG pre-treatment was able to reduce the loss of cell viability induced by the medium of LPS-stimulated macrophages in NSC-34 cells. Indeed, CBG pre-treatment inhibited apoptosis, as shown by the reduction of caspase 3 activation and Bax expression, while Bcl-2 levels increased. Furthermore, CBG pre-treatment counteracted not only inflammation, as demonstrated by the reduction of IL-1β, TNF-α, IFN-γ and PPARγ protein levels assessed by immunocytochemistry, but also oxidative stress in NSC-34 cells treated with the medium of LPS-stimulated RAW 264.7. Indeed, immunocytochemistry showed that CBG pre-treatment reduced nitrotyrosine, SOD1 and iNOS protein levels and restored Nrf-2 levels. All together, these results indicated the neuroprotective effects of CBG, that may be a potential treatment against neuroinflammation and oxidative stress.

## 1. Introduction

Since ancient times, *Cannabis sativa* has been known for its medicinal and psychotropic effects. This plant was discovered to be a reservoir of compounds exerting beneficial properties. Until now about 120 cannabinoids have been isolated from *Cannabis sativa*, including Δ9-tetrahydrocannabinol (Δ9-THC), responsible for the psychotropic effect associated with Cannabis consumption, cannabidiol (CBD) and cannabigerol (CBG) [1]. Other than their psychotropic effects, cannabinoids showed anti-oxidant and anti-inflammatory properties leading to neuroprotection [2,3,4].

Among cannabinoids, CBD is one of the most studied, and its protective effects on different aspects of human health, including in neurodegenerative disorders, are well known [5,6]. On the contrary, our knowledge about CBG, another non-psychoactive phytocannabinoid, is limited, even if the few studies published on CBG showed promising results and encourage the deepening of its effects on human health. We have already demonstrated the CBG antioxidant properties in RAW 264.7 macrophages stimulated with hydrogen peroxide (H_2_O_2_) [7]. Also anti-inflammatory and neuroprotective effects were reported for CBG and its derivatives in vitro and in vivo in neurodegenerative disease models [8,9,10,11,12].

The anti-oxidant and anti-inflammatory actions of CBG are particularly interesting taking into account that both inflammation and oxidative stress play pivotal roles in neurodegeneration [13,14,15]. Indeed, both these processes lead to neuronal cell death, then triggering and amplifying degeneration [16,17]. It is important to consider that the two pathophysiological processes are tightly correlated and influence each other. It is more relevant for the brain that is particularly sensitive to oxidative stress [18]. Indeed, inflammatory cells can produce reactive species at the site of inflammation causing oxidative stress, while reactive oxygen/nitrogen species may initiate an intracellular signaling cascade that induces the expression of pro-inflammatory genes [15,19]. Given the interdependence of these processes, a compound able to act against both inflammation and oxidative stress may be a promising strategy in the treatment of neurodegenerative disorders.

Different cell types participate in the inflammatory process, including macrophages. They act in order to maintain homeostasis and are directly involved in neuroinflammation, when also circulating monocytes are recruited from the periphery and enter into the central nervous system, contributing to the inflammatory process. For these reasons these cells play a pivotal role in central nervous system diseases, such as autoimmune and neurodegenerative diseases [20].

We have already shown the anti-oxidant capacity of CBG in macrophages and in this work, we aimed to deepen our knowledge on CBG properties analyzing its beneficial effects in an in vitro model of neuroinflammation. Specifically, we evaluated whether CBG was able to counteract the toxicity induced in NSC-34 motor neurons by the cell culture medium of (lipopolysaccharide) LPS-stimulated RAW 264.7 macrophages, focusing our attention on the evaluation of CBG anti-inflammatory and anti-oxidant capacities.

## 2. Results

### 2.1. CBG Increased Cell Viability in NSC-34 Motor Neurons

**Figure 1:**
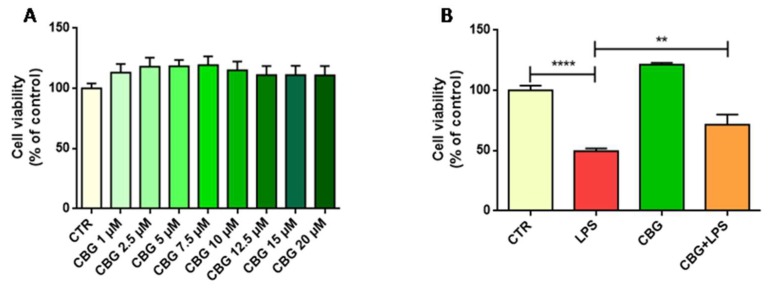
(**A**) Cell viability in NSC-34 motor neurons exposed to different CBG concentrations. All CBG doses increased cell viability compared to the control (CTR). (**B**) Cell viability in NSC-34 exposed to the medium of LPS-stimulated macrophages and in cells pre-treated with CBG. The exposure to the cell culture medium of LPS-stimulated macrophages reduced cell viability, but the pre-treatment with CBG partially restored it. The experiments were performed in triplicate. ** *p* < 0.01 NSC-34 cells treated with the medium of LPS stimulated macrophages vs. NSC-34 cells pre-treated with CBG and then exposed to the medium of LPS stimulated macrophages; **** *p* < 0.0001 NSC-34 treated with the medium of LPS stimulated macrophages vs. control.

In order to evaluate the effects on cell viability of different concentrations of CBG, NSC-34 motor neurons were incubated 24h with the following CBG doses: 1, 2.5, 5, 7.5, 10, 12.5, 15 and 20 µM. We observed with all doses an increase in cell viability compared to the control, even if the difference was not statistically significant. In particular, for doses from 2.5 to 7.5 µM we observed about a 20% increase in cell viability compared to the control. For higher concentrations, cell proliferation decreased even if it was still higher compared to the control (Figure 1A). We included in our analysis also NSC-34 cells incubated with similar concentrations of dimethyl sulfoxide (DMSO) and no cytotoxicity was observed. On the bases of these results, we decided to perform the other experiments with CBG 7.5 µM, because with this concentration we observed the highest increase in cell viability, even if not significant.

### 2.2. CBG Counteracted the Loss of Cell Viability and Inhibited Apoptosis in NSC-34 Cells Treated with the Medium of LPS-Stimulated Macrophages

The incubation with the medium of LPS-stimulated RAW 264.7 macrophages caused a significant loss of cell viability compared to control cells. Indeed, cell viability decreased of about 50% compared to control NSC-34 cells. Interestingly, the pre-treatment with CBG 7.5 µM was able to reduce the loss of cell viability (Figure 1B).

**Figure 2:**
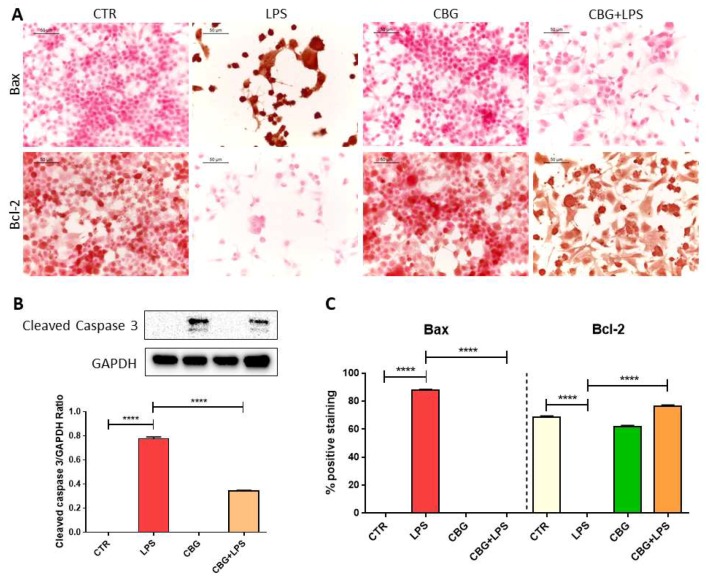
CBG pre-treatment was able to inhibit apoptosis induced by the medium of LPS-stimulated macrophages in NSC-34 motor neurons. (**A**) Immunocytochemistry showed that NSC-34 cells treated with the medium of LPS-stimulated macrophages expressed Bax but not Bcl-2. CBG pre-treatment abolished Bax expression and restored those of Bcl-2. (**B**) The treatment with the medium of LPS-stimulated macrophages induced caspase 3 activation in NSC-34 cells, but CBG pre-treatment reduced its expression. (**C**) Quantitative analysis of positive staining. The experiments were repeated three times. **** *p* < 0.0001, NSC-34 cells treated with the medium of LPS stimulated macrophages vs. NSC-34 pre-treated with CBG and then exposed to the medium of LPS stimulated macrophages, NSC-34 treated with the medium of LPS stimulated macrophages vs. control; Scale bar: 50 µm.

Accordingly, we observed the induction of apoptosis in NSC-34 motor neurons exposed to the medium of LPS-treated macrophages, as demonstrated by the significant increase of the protein levels of cleaved caspase 3 and Bax, while Bcl-2 expression was reduced (Figure 2). On the contrary the pre-treatment with CBG inhibited apoptosis, abolishing the increase in Bax level and reducing cleaved caspase 3, while Bcl-2 expression increased (Figure 2). Control cells and NSC-34 treated with CBG alone expressed Bcl-2, but neither Bax nor cleaved caspase 3.

### 2.3. CBG Reduced the Expression of Pro-Inflammatory Cytokines and Proliferator-Activated Receptor γ (PPARγ)

**Figure 3:**
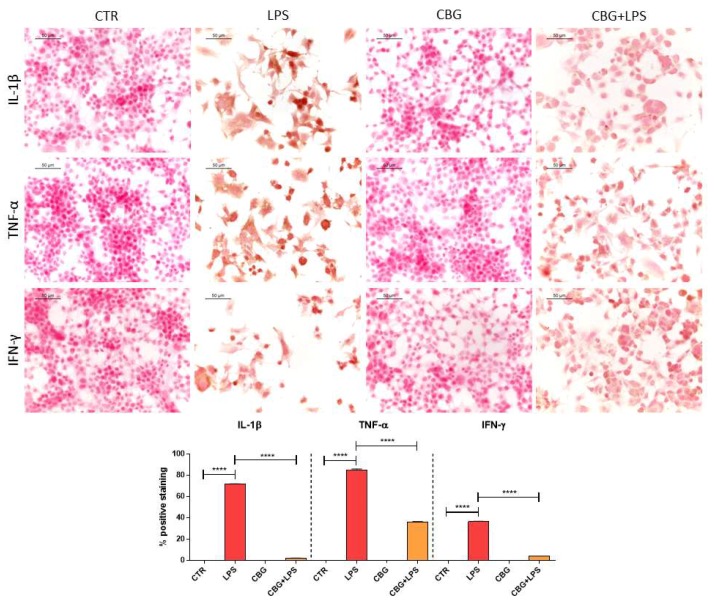
CBG pre-treatment was able to reduce the levels of pro-inflammatory cytokines in NSC-34 cells treated with the medium of LPS-stimulated RAW 264.7 macrophages. Immunocytochemistry with the quantitative analysis of positive staining showed that the treatment with the medium of LPS-stimulated RAW 264.7 macrophages induced the expression of the pro-inflammatory cytokines IL-1β, TNF-α and IFN-γ. CBG pre-treatment reduced the protein levels of the pro-inflammatory cytokines. The immunocytochemical assays were repeated three times. **** *p* < 0.0001, NSC-34 cells treated with the medium of LPS stimulated macrophages vs. NSC-34 pre-treated with CBG and then exposed to the medium of LPS stimulated macrophages, NSC-34 treated with the medium of LPS stimulated macrophages vs. control; Scale bar: 50 µm.

The treatment of NSC-34 motor neurons with the medium of LPS-stimulated macrophages induced inflammation as demonstrated by the increased protein levels of the pro-inflammatory cytokines interleukin-1β (IL-1β), tumor necrosis factor α (TNF-α) and interferon-γ (IFN-γ) evaluated by immunocytochemical assay (Figure 3). Interestingly, the pre-treatment with CBG was able to reduce IL-1β, TNF-α and IFN-γ protein levels (Figure 3). Control and CBG treated NSC-34 motor neurons did not express pro-inflammatory cytokines.

**Figure 4:**
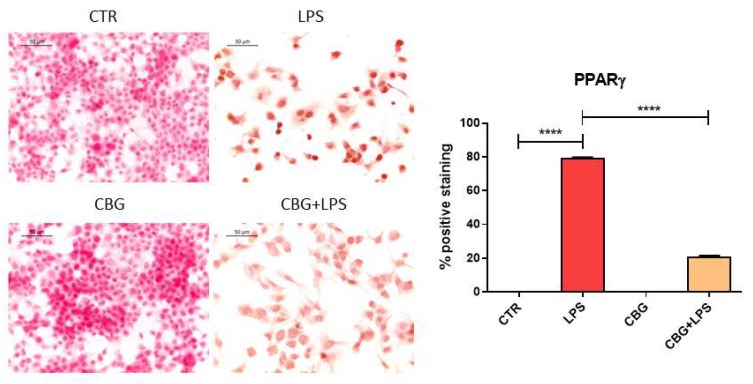
CBG pre-treatment was able to reduce the levels of PPARγ in NSC-34 cells treated with the medium of LPS-stimulated RAW 264.7 macrophages. The treatment with the medium of LPS-stimulated RAW 264.7 macrophages induced the expression of PPARγ, but CBG pre-treatment reduced its levels. The immunocytochemical assays were repeated three times. **** *p* < 0.0001, NSC-34 cells treated with the medium of LPS stimulated macrophages vs. NSC-34 cells pre-treated with CBG and then exposed to the medium of LPS stimulated macrophages, NSC-34 cells treated with the medium of LPS stimulated macrophages vs. control; Scale bar: 50 µm.

In addition, in NSC-34 motor neurons treated with the medium of LPS-stimulated RAW 264.7 cells, we observed the expression of PPARγ assessed by immunocytochemistry. However, the pre-treatment with CBG reduced PPARγ protein levels (Figure 4).

### 2.4. CBG Exerted an Antioxidant Action in NSC-34 Cells Treated with Medium of LPS-Stimulated Macrophages

**Figure 5:**
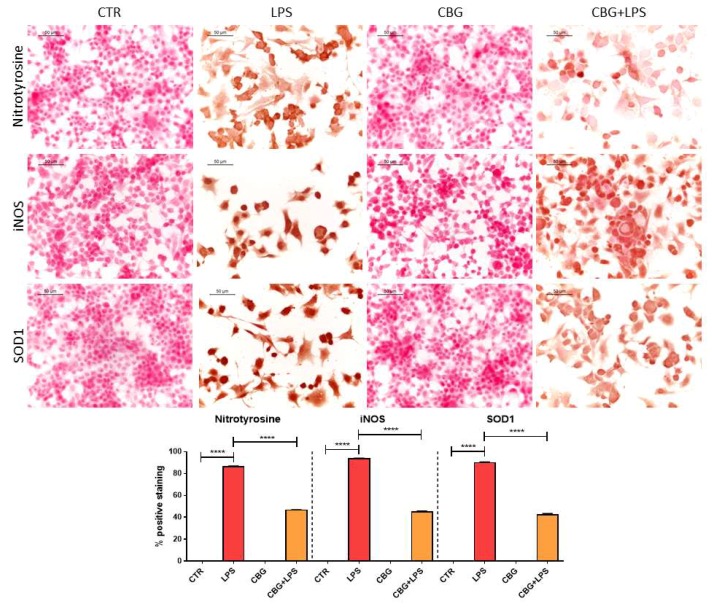
CBG pre-treatment was able to reduce the levels of oxidative stress markers nitrotyrosine, iNOS and SOD1 in NSC-34 cells treated with the medium of LPS-stimulated RAW 264.7 macrophages. The treatment with the medium of LPS-stimulated RAW 264.7 macrophages induced the expression of nitrotyrosine, iNOS and SOD1, but CBG pre-treatment reduced their levels. The immunocytochemical assays were repeated three times. **** *p* < 0.0001, NSC-34 cells treated with the medium of LPS stimulated macrophages vs. NSC-34 cells pre-treated with CBG and then exposed to the medium of LPS stimulated macrophages, NSC-34 cells treated with the medium of LPS stimulated macrophages vs. control; Scale bar: 50 µm.

As we said above, inflammation and oxidative stress are two correlated processes, indeed the treatment of NSC-34 motor neurons with the medium of LPS-stimulated RAW 264.7 induced oxidative stress, as demonstrated by the increase in nitrotyrosine levels evaluated by immunocytochemical assay. However, CBG pre-treatment was able to reduce nitrotyrosine expression (Figure 5). In addition, CBG pre-treatment was able to counteract the increase of superoxide dismutase 1 (SOD1) and inducible nitric oxide synthase (iNOS) expression induced by the treatment with the medium of LPS-stimulated RAW 264.7, reducing their levels (Figure 5) as evidenced by immunocytochemistry. Control NSC-34 motor neurons and cells treated with CBG alone evidenced the absence of oxidative marker expression.

**Figure 6:**
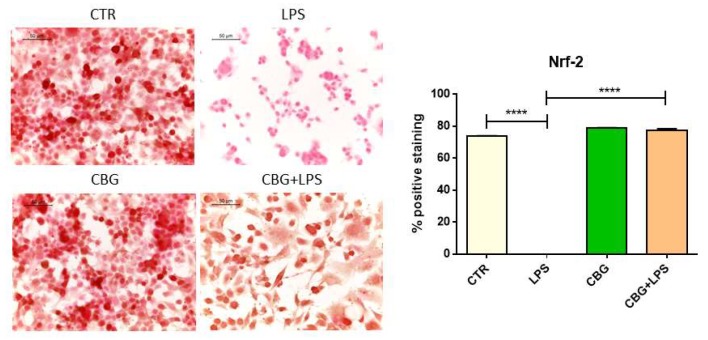
CBG pre-treatment was able to restore Nrf-2 expression in NSC-34 cells treated with the medium of LPS-stimulated RAW 264.7 macrophages. Control cells and NSC-34 cells treated with CBG alone expressed Nrf-2. On the contrary, the treatment with the medium of LPS-stimulated RAW 264.7 macrophages abolished its expression, but CBG pre-treatment restored its levels. The immunocytochemical assays were repeated three times. **** *p* < 0.0001, NSC-34 cells treated with the medium of LPS stimulated macrophages vs. NSC-34 cells pre-treated with CBG and then exposed to the medium of LPS stimulated macrophages, NSC-34 cells treated with the medium of LPS stimulated macrophages vs. control; Scale bar: 50 µm.

Controls and NSC-34 motor neurons exposed to CBG expressed nuclear factor erythroid 2–related factor 2 (Nrf-2). The exposure of NSC-34 motor neurons to the medium of LPS-stimulated macrophages reduced the levels of Nrf-2 (Figure 6). However, CBG pre-treatment restored Nrf-2 levels.

## 3. Discussion

Both oxidative stress and inflammation play a main role in neurodegenerative disorders, including Alzheimer’s disease, Parkinson’s disease and multiple sclerosis [13,14]. Neurodegeneration represents a major problem for human health, being one of the major causes of mortality and disability. Given that a cure against neurodegenerative disorders is not available, the research is focused on the discovery of new compounds able to exert a beneficial action against neurodegeneration, protecting neuronal cells, and able to stop or delay the progression of this kind of diseases. In particular, compounds with both anti-inflammatory and anti-oxidative actions may represent a successful strategy. Different phytochemicals were shown to exert a protective action, and, among these ones, some cannabinoids extracted from *Cannabis sativa* were studied for their beneficial properties. Even if CBD is one of the most studied non-psychoactive phytocannabinoids, CBG was reported to be able to exert different beneficial actions.

In a previous work we showed that CBG reduced oxidative markers, such as iNOS, nitrotyrosine and Poly (ADP-ribose) polymerase 1 (PARP-1) and increase cell anti-oxidant defense through the modulation of SOD1. We reported that CBG anti-oxidant action depends on the CB2 receptors. Moreover, CBG treatment prevented IκB-α phosphorylation and translocation of the nuclear factor-κB (NF-κB) and modulated the mitogen-activated protein (MAP) kinases pathway. All these actions resulted in an inhibition of cell death [7]. 

Other than the anti-oxidant action, CBG showed neuroprotective effects in experimental models of Huntington’s disease [12] and beneficial actions in a model of inflammatory bowel disease [8]. Furthermore, CBG derivatives showed also neuroprotective effects in models of Parkinson’s disease [11], Huntington’s disease [21] and multiple sclerosis [9,10]. 

In this work, we evaluated the beneficial properties exerted by CBG in an in vitro model of neuroinflammation. With this aim, we exposed NSC-34 motor neurons to the cell culture medium of LPS-stimulated RAW 264.7 macrophages.

We observed that CBG increased the number of viable cells compared to the control at all concentrations tested. The highest cell viability was observed at the dose of CBG 7.5 µM, and this concentration was used for further evaluations.

Incubation with the medium of LPS-stimulated RAW 264.7 macrophages caused a 50% loss of cell viability in NSC-34 motor neurons. This result is in line with a study reporting that LPS treatment induced in PC12 rat pheochromocytoma cells, an in vitro model used for neurological and neurochemical studies, loss of cell viability [22]. Interestingly, CBG was able to decrease neuronal cell loss. These results are in line with those obtained with a CBG derivative in M-213 neuronal cells exposed to the conditioned medium of LPS stimulated BV2 cells, in which it was able to reduce M-213 cell death [11]. In addition, the capacity of CBG and of its quinone derivatives to counteract the loss of cell viability induced by neurotoxic stimuli, was also reported in HT22 mouse hippocampal cells and Neuro-2a neuroblastoma cells treated with glutamate [9,21].

Accordingly to the loss of cell viability, the medium of LPS-stimulated macrophages induced apoptosis in NSC-34 motor neurons, as demonstrated by the expression of cleaved caspase 3 and Bax, while Bcl-2 was not expressed. We evaluated cleaved caspase 3 protein level by western blot analysis because this assay better evidenced the presence of the cleaved bands. However, CBG pre-treatment decreased the protein levels of active caspase 3 and Bax was not expressed, instead Bcl-2 levels increased. Similar results were obtained in RAW 264.7 treated with H_2_O_2_, where it was shown that CBG inhibited apoptosis [7].

The treatment of NSC-34 motor neurons with the medium of LPS-stimulated RAW 264.7 increased the expression of the pro-inflammatory cytokines as already reported by previous works [23,24] and in line with the pro-inflammatory role of LPS. CBG pre-treatment was able to reduce inflammation, decreasing the expression of the pro-inflammatory cytokines IL-1β, TNF-α and IFN-γ. CBG capacity to counteract the release of cytokines was reported in other models. Indeed, in an experimental model of inflammatory bowel disease, CBG reduced IL-1β and IFN-γ levels in inflamed colons [8], while in a model of Huntington’s disease, CBG was able to decrease the expression of TNF-α and interleukin 6 (IL-6) [12]. Furthermore, CBG derivatives showed anti-inflammatory properties, such as CBG quinone derivatives that reduced the release of TNF-α and IL-1β in the medium of LPS-treated BV2 cells [11] and the expression of TNF-α and IFN-γ in the spinal cord of a murine model of Experimental Autoimmune Encephalomyelitis (EAE) [10]. Both CBG and its quinone derivative were able to inhibit IL-1β, TNF-α and IL-6 in microglia stimulated with LPS [9]. In our experimental condition, we found that the treatment of NSC-34 motor neurons with the medium of LPS-stimulated RAW 264.7 increased the expression of PPARγ, but CBG pre-treatment reduced its level. PPARγ plays a crucial role in the regulation of proliferation, metabolism, differentiation and inflammatory response in the nervous system. PPARγ agonist can exert anti-inflammatory and anti-oxidant responses [25]. PPARγ was reported to increase in vivo after LPS injection [26,27]. It is known that some cannabinoids can activate PPARγ, that mediates at least in part the analgesic, neuroprotective and anti-inflammatory effects [28]. CBG derivatives exerted neuroprotective, pro-survival and anti-inflammatory actions, at least in part, activating PPARγ [10,11,21].

The incubation with the medium of LPS-stimulated RAW 276.7 increased oxidative stress in NSC-34 cells as demonstrated by the increase in nitrotyrosine levels, a well-known marker of oxidative stress involved also in neurodegenerative disease [29,30]. In parallel, we observed increased levels of iNOS in NSC-34 cells treated with the medium of LPS-stimulated RAW 264.7 macrophages. NOS enzymes mediate the synthesis of nitric oxide (NO) from the conversion of the amino acid l-arginine to l-citrulline. NO can interact with superoxide anion to form peroxynitrite, a potent oxidant agent. It is known that iNOS expression may be induced by both LPS and cytokines [31], such as in our study. However, the pre-treatment with CBG reduced both nitrotyrosine and iNOS expression in NSC-34 motor neurons treated with the medium of LPS-stimulated RAW 264.7 macrophages. CBG ability to reduce the levels of these pro-oxidant markers was demonstrated in vitro in macrophages stimulated with H_2_O_2_ [7]. However, also in in vivo models of Huntington’s disease and experimental inflammatory bowel disease CBG attenuated the expression of iNOS [8,12]. CBG derivatives were reported to decrease iNOS expression in vivo and in vitro [11]. We observed increased SOD1 levels in NSC-34 motor neurons treated with the medium of LPS-stimulated RAW 264.7 macrophages. SOD1 protects cells from harmful amounts of superoxide anions, converting two superoxide anions into oxygen and H_2_O_2_. The pre-treatment with CBG reduced SOD1 levels.

Decreased levels of the transcription factor Nrf-2 in NSC-34 motor neurons exposed to the medium of LPS-stimulated RAW 276.7 macrophages were found. Nrf-2 takes part in the cell anti-oxidant defense system, being a regulator of the expression of genes involved in the protection against oxidative stress and inflammation in order to maintain mitochondrial function, cellular redox, and protein homeostasis [32]. Indeed, among Nrf-2 target genes there are those encoding for proteins involved in detoxification, antioxidant and anti-inflammatory actions [33]. Interestingly, we observed that the pre-treatment with CBG restored Nrf-2 nuclear protein expression reducing oxidative stress. The antioxidant action of CBG is particularly important taking into account that the brain is particularly sensitive to changes in cellular redox status, making the maintenance of redox homeostasis in the brain a critical point for the prevention of oxidative stress induced cellular damage [34,35]. However, Nrf-2 showed also an anti-inflammatory action, given that evidence showed a mechanism of transcriptional repression of pro-inflammatory cytokines, such as TNF-α, IL-1β, IL-6, interleukin 8 in microglia, macrophages, monocytes, and astrocytes following Nrf-2 activation [36,37]. Interestingly, in our in vitro model of neuroinflammation we observed in parallel to the increased Nrf-2 levels, a reduction of pro-inflammatory cytokines.

Our results are in line with previous studies that showed the beneficial effects and anti-inflammatory activity of CBG and its derivatives. In a model of colitis induced in mice by intracolonic administration of dinitrobenzene sulphonic acid (DNBS), CBG was able to reduce colon weight/colon length ratio, myeloperoxidase activity and exerts an anti-inflammatory activity associated to DNBS administration. In addition, CBG reduced NO production and iNOS protein expression in macrophages [8]. CBG showed neuroprotective effects in a Huntington’s disease model, improving motor deficits and reducing microgliosis and inflammatory markers [12]. A derivative of CBG, the CBG quinone VCE-003 showed neuroprotective actions in experimental models of multiple sclerosis [9,10]. Indeed, VCE-003 mitigated disease symptoms, decreased microglia reactivity and modulated the expression of genes involved in multiple sclerosis pathology. In addition, VCE-003 showed anti-inflammatory properties, protecting neurons from excitotoxicity and inhibiting the release of pro-inflammatory mediators in LPS stimulated microglial cells. Another CBG derivative, VCE-003.2 prevented neuronal degeneration in an experimental model of Parkinson’s disease, through an anti-inflammatory action [11].

It would be interesting also to perform gene expression analysis, in order to evaluate the transcriptional regulation exerted by CBG. However, we only evaluated protein levels, and then this may be considered a limitation of this study. In addition, it is an in vitro study, then these results have to be confirmed in vivo in a neuroinflammation model, but also in different neurodegenerative disorder models.

## 4. Materials and Methods 

### 4.1. Plant Material

*Cannabis sativa* var. Carma was obtained from a greenhouse cultivation at CREA-CIN (Rovigo, Italy), where a voucher specimen is kept. The plant material was harvested in November 2010 and was supplied by Dr. Gianpaolo Grassi (CREA, Rovigo, Italy). The manipulation of the plant was done in accordance with its legal status (Authorization SP/101 of the “Ministerodella Salute”, Rome, Italy).

### 4.2. General Experimental Procedures

^1^H NMR spectra were measured using JEOL ECP 300—300 MHz spectrometer (JEOL, Pleasanton, CA, USA). Chemical shifts were referenced to the residual solvent signal (CDCl_3_: δH 7.26). Reverse phase (RP) C-18 (POLYGOPREP60-30 C18) was used to remove waxes and pigments. Silica gel 60 (70–230 mesh) was used for gravity column chromatography. CBG purification was monitored using TLC on Merck 60 F254 (0.25 mm) plates, that were visualized by UV inspection and/or spraying with 5% H_2_SO_4_ in ethanol and heating.

### 4.3. Extraction and CBG Isolation

One Kg of dried, powdered flowered aerial parts were heated at 120°C for 2.5 h in order to decarboxylate precannabinoids and then extracted exhaustively with acetone (2 × 9 L) using a shaker. The elimination of the solvent left a black resinous residue (74 g, 7.4%), that was dissolved in MeOH (30 mL/g of extract) and filtered using RP C-18 silica gel to remove waxes and pigments.

The evaporation of methanol afforded 36 g of a dark-green extract that was further purified by gravity column chromatography on silica gel (75 g, petroleum ether-EtOAc, 8:2, as eluent) to afford 5 g of a yellow oil then crystallized with petroleum ether to give 3 g of pure CBG (0.3%). Pure CBG was stored at −8 °C.

### 4.4. RAW 264.7 Macrophage Cell Culture and LPS Treatment

The murine macrophage RAW 264.7 cell line, obtained from InvivoGen (San Diego, CA, USA), were cultured in monolayer at 37 °C in a moisturized atmosphere of 5% CO_2_ and 95% air using DMEM-high glucose medium (Sigma-Aldrich,St. Louis, MO, USA supplemented with 10% fetal bovine serum (FBS) (Sigma-Aldrich). With the aim to induce inflammation, cells were grown until 70–80% confluence was reached, and after they were incubated with 10 ng/mL LPS from Escherichia coli 0111:B4 (Sigma-Aldrich) for 24 h [38]. Untreated cells were used as control. At the end of the treatment the culture medium was collected to carry out experiments with NSC-34 cells.

### 4.5. NSC-34 Motor Neurons Treatment with the Medium of LPS-Stimulated RAW 264.7

The aim of this work was the evaluation of the anti-inflammatory and anti-oxidative stress properties of CBG in a neuroinflammation model in vitro. In order to reproduce neuroinflammation in vitro, we treated the murine motor neuron cell line NSC-34 with the cell culture medium of LPS-stimulated RAW macrophages. NSC-34 cells, acquired from Cellutions Biosystems Inc., Cedarlane (Burlington, ON, Canada), were cultured in DMEM-high glucose medium (Sigma-Aldrich) with the addition of 10% FBS (Sigma-Aldrich), at 37 °C in a moisturized atmosphere of 5% CO_2_ and 95% air. To examine anti-inflammatory effects of CBG against LPS-induced inflammation, NSC-34 cells were pre-treated for 24 h with 7.5 µM CBG. At the end of pre-treatment, medium was replaced with cell-free conditioned medium from LPS-stimulated RAW 264.7 macrophages, and incubated for 24 h. As controls, NSC-34 cells were incubated with the medium of unstimulated RAW 264.7 macrophages. Cells treated with vehicle (<0.1% DMSO) or with the CBG alone were also included as controls. Then, motor neuronal cells were fixed for immunocytochemistry analysis or harvested for western blot. All the experiments were done in triplicate and repeated for three independent times.

### 4.6. Thiazolyl Blue Tetrazolium Bromide (MTT) Assay

In order to evaluate the effects of CBG and LPS on cell viability the MTT assay was performed. To evaluate the effects of CBG, NSC-34 cells were cultured in 96-well plates and incubated for 24 h with different CBG doses (1, 2.5, 5, 7.5, 10, 12.5, 15 and 20 µM). With the aim to evaluate cell death induced by LPS and whether CBG could counteract the loss of cell viability, NSC-34 motor neurons were cultured in 96-well plates, and after pre-treatment with 7.5 µM CBG for 24 h, cells were incubated with the medium of LPS-stimulated macrophages for 24 h. At the end of the treatments, cells were washed and incubated with fresh medium containing MTT (0.5 mg/mL; Sigma-Aldrich) at 37°C for 4 h. After, formazan crystals were dissolved in acidic isopropanol at 37°C for 1 h and the optical density was evaluated by spectrophotometric measurement of absorbance. All the experiments were done in triplicate and repeated for three independent times.

### 4.7. Protein Extraction and Western Blot Analysis

At the end of the treatment, NSC-34 motor neurons were collected and lysed using buffer A [320 mM sucrose, 10 mM, 1 mM EGTA, 2 mM EDTA, 5 mM NaN3, 50 mM NaF, β-mercaptoethanol, and protease/phosphatase inhibitor cocktail (Roche Molecular Diagnostics, Branchburg, NJ, USA)] in ice for 15 min, and centrifuged at 1000×*g* for 10 min at 4 °C. The supernatant was collected as cytosolic extract. The obtained pellet was lysed with buffer B [150 mM NaCl, 10 mM Tris-HCl (pH 7.4), 1 mM EGTA, 1 mM EDTA, Triton X-100, and protease/phosphatase inhibitor cocktail (Roche Molecular Diagnostics, Pleasanton, CA, USA)] in ice for 15 min and centrifuged at 15,000×*g* for 30 min at 4 °C. The supernatant was collected as nuclear extract. Protein concentrations were measured through Bradford assay (Bio-Rad Laboratories, Inc., Hercules, CA, USA). Twenty-five μg of proteins were heated for 5 min at 95 °C and resolved by SDS-polyacrylamide gel electrophoresis (SDS-PAGE) and after transferred onto a PVDF membrane (Amersham Hybond, GE Healthcare Life Sciences, Milan, Italy).

Membranes were blocked with 5% skim milk in Phosphate Buffered Saline (PBS) for 1 h at room temperature and incubated overnight at 4 °C with the primary antibody against cleaved caspase 3 (1:1000; Cell Signaling Technology, Danvers, MA, USA). Membranes were washed with PBS 1X and incubated with horse radish peroxidase (HRP)-conjugated anti-rabbit IgG secondary antibody (1:2000; Santa Cruz Biotechnology,Dallas, TX, USA) for 1 h at room temperature. With the aim to analyze if blots were loaded with equal amounts of proteins, membranes were incubated with antibody for glyceraldehyde 3-phosphate dehydrogenase (GAPDH) HRP Conjugated (1:1000; Cell Signaling Technology). The relative expression of protein bands was analyzed through an enhanced chemiluminescence system (Luminata Western HRP Substrates; Millipore, Burlington, MA, USA). Protein bands were acquired using ChemiDoc™ MP System (Bio-Rad Laboratories, Inc.) and quantified through the computer program ImageJ software (developed at the National Institutes of Health, USA, http://rsb.info.nih.gov/ij). All blots are representative of three independent experiments.

### 4.8. ImmunocytoChemistry

NSC-34 motor neurons were grown on coverslips (12 mm; Thermo Fisher Scientific, Waltham, MA, USA) and, at the end of the treatments, they were fixed with 4% paraformaldehyde at room temperature for 30 min and after washed with PBS (pH 7.5). Afterwards, cells were incubated with 3% H_2_O_2_ at room temperature for 15 min in order to suppress the endogenous peroxidase activity. After three washes with PBS, cells were blocked with horse serum +0.1% Triton X-100 for 20 min and incubated overnight at 4°C with the following primary antibodies:

- anti Bax (1:50; Santa Cruz Biotechnology);
- anti Bcl-2 (1:50; Santa Cruz Biotechnology);
- anti IL-1β (1:250; Cell Signaling Technology);
- anti IFN-γ (1:50; Santa Cruz Biotechnology);
- anti TNF-α (1:250; Cell Signaling Technology);
- anti SOD1 (1:100; Abcam, Cambridge, UK);
- anti iNOS (1:50; Santa Cruz Biotechnology);
- anti nitrotyrosine (1:1000; Millipore);
- anti Nrf-2 (1:50; Santa Cruz Biotechnology);
- anti PPARγ (1:50; Santa Cruz Biotechnology).

After, cells were washed with PBS and incubated with biotinylated secondary antibody (1:200; Vector Laboratories, Inc., Burlingame, CA, USA) and streptavidin AB Complex-HRP (ABC-kit from Dako, Glostrup, Denmark). The immunostaining was developed with the DAB peroxidase substrate kit (Vector Laboratories, DBA Italia S.r.l., Milan, Italy; brown color; positive staining) and counterstaining with nuclear fast red (Vector Laboratories, DBA Italia S.r.l.; pink background; negative staining).

The immunocytochemical assays were repeated three times and each experimental group was plated in duplicate, for a total of 6 coverslips for each antibody. With the aim of calculating the percentage of positive cells stained, the images were captured using a light microscopy (LEICA DM 2000 combined with LEICA ICC50 HD camera) with an objective ×40 and the densitometric analysis was carried out using the software LEICA Application Suite ver. 4.2.0 (LEICA, Wetzlar, Germany). Quantitative analysis was performed on 6 coverslips by covering approximately 90% of the total area.

### 4.9. Statistical Data Analysis

Statistical analysis was carried out using GraphPad Prism version 6.0 software (GraphPad Software, La Jolla, CA, USA). The data were statistically analyzed by one-way ANOVA test and Bonferroni post-hoc test for multiple comparisons. A P value less than or equal to 0.05 was considered statistically significant. Results are reported as mean ± SEM of N experiments.

## 5. Conclusions

In conclusion, our results indicated that CBG exerted a protective action in an in vitro neuroinflammation model. CBG reduced neuronal death in NSC-34 cells treated with the medium of LPS stimulated macrophages, reducing inflammation and oxidative stress. In particular, CBG restored cell anti-oxidant defense, increasing the expression of Nrf-2, reduced oxidative stress and inflammatory markers. On the bases of these results, thanks to its neuroprotective effects, we encourage the use of CBG against neurodegeneration and in those pathological conditions where neuroinflammation and oxidative stress play a main role.

## Abbreviations

Δ9-THC	Δ9-tetrahydrocannabinol
CBD	Cannabidiol
CBG	Cannabigerol
H_2_O_2_	Hydrogen peroxide
LPS	Lipopolysaccharide
DMSO	Dimethyl sulfoxide
PPARγ	Proliferator-activated receptor γ
IL-β	Interleukin-1β
IFN-γ	Interferon-γ
TNF-α	Tumor necrosis factor α
iNOS	Inducible nitric oxide synthase
SOD1	Superoxide dismutase1
Nrf-2	Nuclear factor erythroid 2–related factor 2
PARP-1	Poly [ADP-ribose] polymerase 1
NF-κB	Nuclear factor-κB
MAP	Mitogen-activated protein
IL-6	Interleukin 6
EAE	Experimental Autoimmune Encephalomyelitis
NO	Nitric oxide
DNBS	Dinitrobenzene sulphonic acid
FBS	Fetal bovine serum
MTT	Thiazolyl Blue Tetrazolium Bromide
HRP	Horse radish peroxidase
GAPDH	Glyceraldehyde 3 phosphate dehydrogenase
PBS	Phosphate Buffered Saline
